# An international multicenter efficacy and safety study of IqYmune in initial and maintenance treatment of patients with chronic inflammatory demyelinating polyradiculoneuropathy: PRISM study

**DOI:** 10.1111/jns.12408

**Published:** 2020-08-31

**Authors:** Eduardo Nobile‐Orazio, Sonia Pujol, Fabrice Kasiborski, Rabye Ouaja, Gilles Della Corte, Robert Bonek, Dario Cocito, Angelo Schenone

**Affiliations:** ^1^ Neuromuscular and Neuroimmunology Service, IRCCS Humanitas Clinical and Research Center Milan University Via Manzoni 56, Rozzano Milan 20089 Italy; ^2^ LFB Les Ulis France; ^3^ Dellmed Consulting Ornex France; ^4^ Department of Neurology NeuroCenter, Regional Specialist Hospital Bydgoszcz Poland; ^5^ Istituti Clinici Scientifici Maugeri Turin Italy; ^6^ Department of Neurosciences, Rehabilitation, Ophthalmology, Genetic and Maternal and Infantile Sciences (DINOGMI) University of Genova and IRCCS Policlinico San Martino Genova Italy

**Keywords:** chronic inflammatory demyelinating polyradiculoneuropathy, inflammatory neuropathy cause and treatment, intravenous immunoglobulin, IqYmune, PRISM

## Abstract

This prospective, multicenter, single‐arm, open‐label phase 3 study aimed to evaluate the efficacy and safety of IqYmune in patients with chronic inflammatory demyelinating polyradiculoneuropathy (CIDP). Patients received one induction dose of 2 g/kg and then seven maintenance doses of 1 g/kg at 3‐week intervals. The primary endpoint was the responder rate at the end of study (EOS), defined as an improvement of ≥1 point on the adjusted inflammatory neuropathy cause and treatment (INCAT) disability scale. The responder rate was compared with the responder rate of a historical placebo group (33.3%). Secondary endpoints included changes from baseline to EOS of adjusted INCAT disability score, grip strength, Medical Research Council (MRC) sum score, Rasch‐modified MRC sum score, Rasch‐built overall disability scale score and the clinical global impression. Forty‐two patients, including 23 Ig‐naïve and 19 Ig‐pre‐treated, were included in the efficacy set. The overall response rate at EOS was 76.2% (95% confidence interval [60.5%‐87.9%]). The superiority of IqYmune compared to the historical placebo control was demonstrated (*P* < .0001). The responder rate was numerically higher in Ig‐pre‐treated than in Ig‐naïve patients but confidence intervals were overlapping (84.2% [60.4%‐96.6%] vs 69.6% [47.1%‐86.8%]). All secondary endpoints confirmed this conclusion. The median time to response was 15 weeks [8.9‐19.1 weeks]. A total of 156 adverse events including five serious were considered related to IqYmune, 87.2% were mild. Neither hemolysis nor signs of renal or hepatic impairment were observed. These results demonstrate that IqYmune is an effective and well‐tolerated treatment in patients with CIDP.

## 1 INTRODUCTION

Chronic inflammatory demyelinating polyradiculoneuropathy (CIDP) is an acquired rare heterogeneous disorder affecting sensory and motor peripheral nerves caused by a patchy demyelinating process than can produce sensory loss and positive sensory symptoms as well as motor weakness.^1^ The worldwide prevalence is estimated to be 2 to 7 individuals per 100 000.^2^

Published in 2008, the ICE study is considered the reference study for the treatment of CIDP with intravenous immunoglobulin (IVIg), given the high number of evaluated patients (n = 117). This study showed short‐term and long‐term efficacy and safety of a 10% IVIg in this indication.^3^ The favorable benefit‐risk ratio of IVIg in CIDP was confirmed by an additional study.^4^

IqYmune (I10E for code product) is a highly purified, ready‐to‐use, 10% liquid preparation of normal human immunoglobulin (100 mg/mL) for intravenous administration. This formulation is obtained from thousands of healthy donors and has been shown to be effective and well tolerated in patients with primary immunodeficiency^5^ (PID), multifocal motor neuropathy^6^ (MMN), and primary immune thrombocytopenia (ITP).^7^

IqYmune was designed in a novel purification process based on the quality by design (QbD) approach^8^ for eliminating or reducing impurities in order to reduce the occurrence of adverse events (AEs), while maintaining the structural and functional integrity of IgG thereby ensuring a constant batch‐to‐batch product quality based on a clear knowledge of process limits and a justified control strategy.

The phase 3 prospective international safety multinational study (PRISM) study was conducted from early 2015 to late 2017 to obtain the approval of CIDP in Europe. Starting in 2015, IqYmune has received several approvals in Europe as well in non‐European countries (Mexico and Switzerland), as a replacement therapy for PID, MMN, for various types of hypogammaglobinemia, and for immunomodulation in ITP, Guillain‐Barré syndrome, CIDP, and Kawasaki disease.

This study aimed to demonstrate the improvement of disability by IqYmune in adult patients with CIDP previously treated with IVIg or not. The assessment of safety was a secondary objective.

## 2 MATERIALS AND METHODS

### 2.1 Study design and treatment

This study was a phase 3, international, multicenter, single‐arm, open‐label, prospective trial. The study procedures were in accordance with the International Conference on Harmonization Good Clinical Practice guidelines and the Declaration of Helsinki. The study protocol and all other study‐related documents were reviewed and approved by the local Independent Ethics Committees. Written informed consent was obtained from all patients before they started the study. This study is registered at ClinicalTrials.gov (NCT02293460) and EudraCT (2013‐005557‐73). The recruitment started in February 2015 and the last patient terminated the study in September 2017. The study involved 23 active sites in Italy (6), Spain (5), Tunisia (4), France (3), Turkey (2), Poland (2), and UK (1).

IqYmune was administered at an initial dose of 2 g/kg over 2 to 5 days during the first course, then maintenance doses of 1 g/kg over 1 to 2 days repeated every 3 weeks (± 7 days) during the 7 subsequent courses. Allowed infusion rates ranged from 0.5 to 6 mL/kg/h. For patients older than 65 years, the maximum infusion flow rate was not to exceed 2 mL/kg/h. The duration of the treatment was approximately 21 weeks and the entire follow‐up period was 24 weeks as end of the study (EOS) visit occurred 3 weeks after the last treatment course.

### 2.2 Participant selection

Male or female patients aged 18 years or older with definite or probable CIDP according to the European Federation of Neurological Societies (EFNS)/Peripheral Nerve Society (PNS) guidelines 2010 clinical and neurophysiological criteria,^9^ previously treated with IVIg or not, and with a disability score of at least two on the adjusted inflammatory neuropathy cause and treatment (INCAT) at inclusion were eligible for this study. Patients with the following atypical forms could be included: pure motor CIDP (provided that a diagnosis of MMN has been ruled out), pure sensory CIDP, CIDP associated with a monoclonal gammopathy of unknown significance without antibodies to myelin‐associated glycoprotein and Lewis‐Sumner syndrome.

Patients were considered as Ig‐naïve if they had never been previously treated with Ig and relapsing Ig‐pre‐treated when they had been previously treated with Ig but were in clinical relapse after treatment withdrawal. For Ig‐pre‐treated patients, a washout period of at least 3 months prior to screening was required.

Main exclusion criteria included a history of severe allergic reaction or severe adverse reaction to any Ig, IgA deficiency, any cardiovascular condition representing a thrombo‐embolic risk factor, use of loop diuretics, progressive hepatic disease or serum level of aminotransferase enzymes >2 times upper limit of normal, any serious medical conditions preventing the patient from complying with the protocol requirements or interfering with the evaluation criteria of the study. Patients were also excluded in case of treatments interfering with the IVIg efficacy evaluation either used in the previous 3 months (plasma exchange, blood derivatives, high‐dose steroids) or in the previous 12 months (immunomodulatory/immunosuppressant agents or hematopoietic stem cell transplantation) prior to screening.

### 2.3 Outcome measures and response criteria

The primary efficacy endpoint was the responder rate at EOS visit (week 24) based on the adjusted INCAT disability score, a measurement of activity limitation of the upper and lower limbs as used by Hughes^3^ in the pivotal clinical study (ICE) to support the approval of Gamunex in CIDP; the placebo group results of this trial has been used for historical reference. The disability of the arms is scored from 0 to 4 and of the legs from 0 to 5. The total score can vary from 0 (normal) to 9 (maximal disability). The adjusted INCAT score was measured at baseline and every 3 weeks thereafter, until the end of the study (Week 24). Response was defined a decrease of ≥1 point in the adjusted INCAT disability score between baseline and EOS visit or the last study visit in case of premature discontinuation.

Secondary efficacy endpoints either assessing a different approach of the primary endpoint or exploring different aspects of the disability were performed to reinforce the robustness of measurements and to substantiate the efficacy of IqYmune in CIDP patients. These included responder rate at week 12, time to response, changes from baseline to week 12 (not reported in this article) and to EOS visit for adjusted INCAT disability score, and grip strength in both hands with a Martin Vigorimeter,^10^ and other neurological scores for CIDP. These scores, recommended by peripheral neuropathy outcome measurement standardization (PeriNomS) study group,^11^ were Medical Research Council (MRC) 12 muscles sum score, Rasch‐modified MRC sum score and Rasch‐built overall disability scale (R‐ODS) score.

Last, the clinical global impression (CGI) was assessed by the investigator and the patient. CGI consists of three different global measures: the severity of illness, the global improvement, and the efficacy index including therapeutic effect and side effects. The evaluations were performed at week 12 and EOS visit. The severity of illness was also assessed at baseline.

Serum IgG trough level was measured at baseline and every 3 weeks within 24 hours prior to each course of treatment.

The investigator could discontinue a patient from IqYmune administration if the patient's adjusted INCAT disability score increased by one point compared to baseline and the investigator decided to change the treatment (administration of another IVIg, increase, or initiation of a steroid therapy, immunosuppressive therapy, and plasma exchange).

Throughout the study, safety was evaluated by assessing the occurrence of AEs and their relationship to IqYmune. AEs were assessed in terms of percentages of patients affected. A physical examination was performed and vital signs, biochemical, and hematological parameters were also monitored.

### 2.4 Data analyses

The study was designed to demonstrate superiority of IqYmune compared to the historical placebo control estimated from the ICE study.^3^ Thirty‐eight evaluable patients were needed in order to obtain 90% power using an exact binomial test with a 1‐sided at the nominal level of significance of α = 2.5%. This sample size was based on the predefined success criterion of 60% of responders with IqYmune and of 33% with the historical placebo control in the ICE study which was estimated based on upper boundary of the 95% Clopper‐Pearson confidence interval (CI) of the observed rate (12/58 = 20.7%, 95% CI [11.2%‐33.3%]). To note: the responder rate in ICE study for the group treated with IVIg was 54% (32/59 patients including 39 Ig‐naïve and 20 Ig‐pre‐treated) based on the same definition for response. Patients included could be either Ig‐naïve or relapsing Ig‐pre‐treated patients; each subgroup was described but no statistical test has been performed. Analyses for efficacy were performed based on the full analysis set (FAS) which included all patients who received the drug at least once (ie, total treated set [TTS]) and had available assessment of the primary efficacy endpoint. Analyses for safety were performed based on the TTS. Per‐protocol analyses based on the FAS patients without any major protocol deviation (deviations as having a potential impact on the primary efficacy endpoint) were performed. Missing data was handled using the last observation carried forward method. Patients withdrew due to lack of efficacy of IqYmune were classified as non‐responders and INCAT measurements after the intake of prohibited treatment were censored.

For the secondary efficacy endpoints, the change from baseline to week 12 (not presented in this article) and to EOS was analyzed by producing non‐parametric Hodges‐Lehmann point estimates and their corresponding 95% CIs. The time to adjusted INCAT response was analyzed using the Kaplan‐Meier method. Descriptive statistics (mean, SD, median, minimum, maximum, and 25%‐75% quantile) were calculated for all safety variables.

## 3 RESULTS

### 3.1 Patient disposition

**FIGURE 1:**
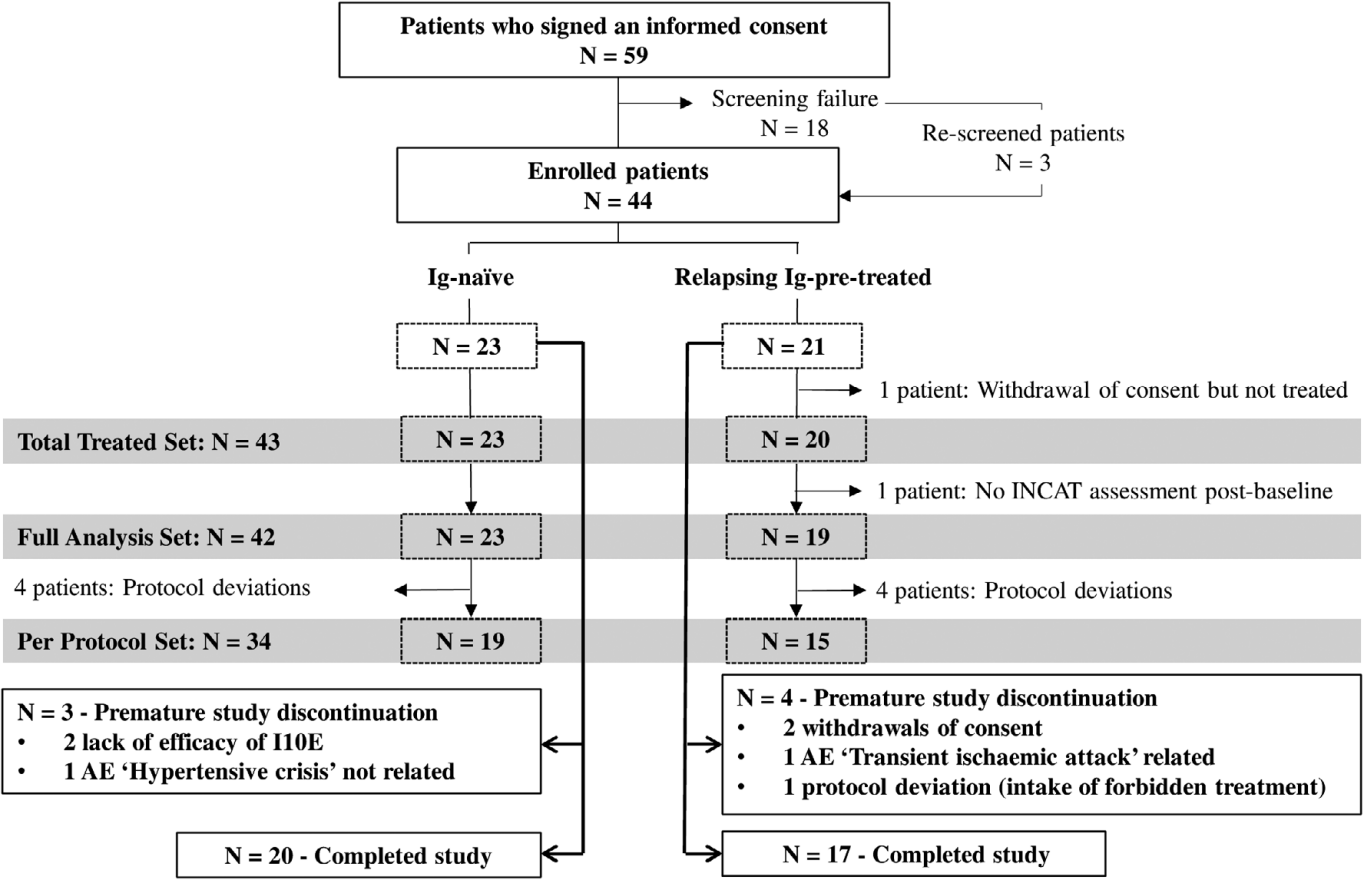
Patient disposition. AE, adverse event; INCAT, inflammatory neuropathy cause and treatment

This study was conducted from February 2015 to March 2017. From 59‐screened patients, a total of 44 patients (23 Ig‐naïve and 21 relapsing Ig‐pre‐treated) were enrolled. Forty‐three patients were included in the TTS. Forty‐two patients with available assessment of the primary efficacy endpoint were included in the FAS and 34 patients were included in the per‐protocol set (PPS). From the 44 patients enrolled, 37 patients completed the 24‐week treatment period. Disposition of the patients and reasons for premature study discontinuation are given in Figure 1.

Table 1 shows patient demographics and baseline characteristics. Forty patients (93%) had definite and 3 (7%) had probable CIDP; 37 patients (86%) had typical form of CIDP and six patients (14%) had atypical form. The mean time since first symptoms was comparable between Ig‐naïve and Ig‐pre‐treated patients. Although no statistical tests were performed to compare both subgroups, the mean age, time since diagnosis, and total adjusted INCAT disability score seemed numerically higher in Ig‐pre‐treated patients than Ig‐naïve patients.

**TABLE 1:** Demographics and disease characteristics patients at baseline ‐ TTS

	Ig‐naïveN = 23	Ig‐pre‐treatedN = 20	OverallN = 43
Age (years)			
Mean ± SD	48.7 ± 15.8	55.8 ± 15.0	52.0 ± 15.7
Median	48.0	59.0	50.0
Min‐max	21‐78	24‐79	21‐79
Gender, N (%)			
Male	12 (52.2%)	12 (60.0%)	24 (55.8%)
Female	11 (47.8%)	8 (40.0%)	19 (44.2%)
Body mass index (kg/m^2^)			
Mean ± SD	25.6 ± 4.8	24.3 ± 4.4	25.0 ± 4.6
Median	25.4	24.6	25.1
Min‐max	18.2‐40.0	14.3‐31.9	14.3‐40.0
Diagnostic categories of CIDP, N (%)^a^			
Definite	20 (87%)	20 (100%)	40 (93%)
Probable	3 (13%)	0	3 (7%)
Time since first symptoms (years)			
Mean ± SD	6.7 ± 7.4	6.7 ± 5.6	6.7 ± 6.5
Median	3.6	3.4	3.4
Min‐max	0.4‐28.6	0.4‐17.3	0.4‐28.6
Time since diagnosis (years)			
Mean ± SD	2.7 ± 6.0	5.0 ± 5.5	3.8 ± 5.8
Median	0.2	2.4	1.2
Min‐max	0.0^b^‐23.3	0.0^c^‐16.8	0.0^c^‐23.3
Total adjusted INCAT disability score			
Mean ± SD	2.7 ± 1.3	3.3 ± 1.8	3.0 ± 1.5
Median	2.0	2.5	2.0
Min‐max	2‐6	2‐8	2‐8

*Note*: Range of total adjusted INCAT disability score: 0 (normal) to 9 (maximum disability). Range of adjusted INCAT disability arms score: 0 to 4. Range of adjusted INCAT disability legs score: 0 to 5.

Abbreviations: CIDP, chronic inflammatory demyelinating polyneuropathy; INCAT, inflammatory neuropathy cause and treatment; N, number of patients; TTS, total treated set.

^a^ CIDP diagnosis was considered as definite or probable according to the EFNS/PNS guidelines 2010 clinical and neurophysiological criteria.

^b^ The minimal values of 0.0 years correspond to periods of time < 18 days (since Min values were documented in year with only one decimal).

^c^ In the Ig‐pre‐treated subgroup, one patient had been pre‐treated 5 months before by Ig for CIDP symptoms, but the diagnosis was confirmed only 4 days before the inclusion in the study explaining the value 0.0 year for time since diagnosis.

### 3.2 Study treatment administration

The 43 patients of the TTS received total of 319 courses administered as 581 infusions.

Overall, patient exposure was similar between Ig‐naïve and Ig‐pre‐treated patients (supplementary information Table S1).

Theoretically, a patient who fully completed the study should have IqYmune exposure of 6 months, a total number of courses of eight, a total number of infusions from 9 to 19 and a maximal flow rate of 6 mL/kg/h. Both actual mean duration of exposure (5.3 ± 1.4 months) and total number of courses (7.4 ± 1.8) are close to the theoretical values confirming the good compliance. The flow rate did not exceed 6 mL/kg/h during the study.

### 3.3 Efficacy

#### 3.3.1 Primary efficacy endpoint

At the end of the study visit, 32 (76.2%) of 42 patients in the FAS were responders, with the two‐sided 95% Exact Clopper‐Pearson CI of [60.5%‐87.9%] (Table 2). The *P*‐value of the one‐sided exact binomial Clopper‐Pearson test (*P* < .0001) indicates that the response rate is statistically higher than the higher bound of the 95% CI (33.3%) of the historical placebo control group in the ICE study.

**TABLE 2:** Primary efficacy endpoint‐responder rate at EOS visit‐FAS

	Ig‐naïveN = 23	Ig‐pre‐treatedN = 19	OverallN = 42
Number of responders (%)^a^	16 (69.6)	16 (84.2)	32 (76.2)
95% Confidence interval^d^	47.1; 86.8	60.4; 96.6	60.5; 87.9
*P*‐value^b^	–	–	<0.0001
Number of non‐responders (%)^c^	7 (30.4)	3 (15.8)	10 (23.8)

Abbreviations: EOS, end of study; FAS, full analysis set; INCAT, inflammatory neuropathy cause and treatment; N, number of patients.

^**a**^ Responders were defined as patients with a decrease of ≥1 point in the adjusted INCAT disability score between baseline and EOS visit (week 24) or the last study visit in case of premature discontinuation.

^b^ one‐sided Exact Clopper‐Pearson (exact binomial test) against the historical placebo response rate of 33.3%.

^c^ A patient was also considered as a non‐responder if the patient was early withdrawn due to lack of efficacy of the treatment (ie, insufficient response to IqYmune).

^d^ Two‐sided Exact Clopper‐Pearson 95% confidence interval.

Although no statistical tests were performed to compare both subgroups, the rate of response would appear higher in the Ig‐pre‐treated subgroup than in the Ig‐naïve subgroup but CIs of these subgroups were largely overlapping (84.2% with a 95% CI of [60.4%‐96.6%] vs 69.6% [47.1%‐86.8%]). Note that the lower bound of the 95% CI was also greater than 33.3% for both subgroups.

Results of the primary endpoint based on the PPS supported the primary analysis, 25 (73.5%) of 34 patients were responders with 95% Exact Clopper‐Pearson CI of [55.6%‐87.1%] (*P* < .0001). In addition, the responder rate at week 12 was 47.6%. Compared to the 76.6% responder rate at the end of the study, it is important to note that 29% of the patient had achieved a response after the fifth course. In other words, 12 responders (three Ig‐pre‐treated patients and nine Ig‐naïve patients) out of 32 showed a late response. Three patients had achieved a response at week 15, three at week 21, four at week 18, and two at EOS visit as shown in Table 3.

**TABLE 3:** Earliest visit with response achieved for responders‐FAS

	Number (%) of patients		
Time points	Ig‐naïveN = 16	Ig‐pre‐treatedN = 16	OverallN = 32
Week 3‐before the second course	2 (12.5%)	5 (31.3%)	7 (21.9%)
Week 6‐before the third course	2 (12.5%)	4 (25.0%)	6 (18.8%)
Week 9‐before the fourth course	2 (12.5%)	3 (18.8%)	5 (15.6%)
Week 12‐before the fifth course	1 (6.3%)	1 (6.3%)	2 (6.3%)
Week 15‐before the sixth course	2 (12.5%)	1 (6.3%)	3 (9.4%)
Week 18‐before the seventh course	3 (18.8%)	1 (6.3%)	4 (12.5%)
Week 21‐before the eighth course	2 (12.5%)	1 (6.3%)	3 (9.4%)
Week 24 (EOS)‐after the eighth course	2 (12.5%)	0	2 (6.3%)

Abbreviations: EOS, end of study; FAS, full analysis set.

#### 3.3.2 Secondary efficacy endpoints

##### Time to response

Time to response was estimated using a subset of patients with response at EOS visit. Non‐responder patients were considered censored at their last assessment of the adjusted INCAT disability score. Patients who took a prohibited treatment before a response were considered censored at the date of the first intake of prohibited treatment.

**FIGURE 2:**
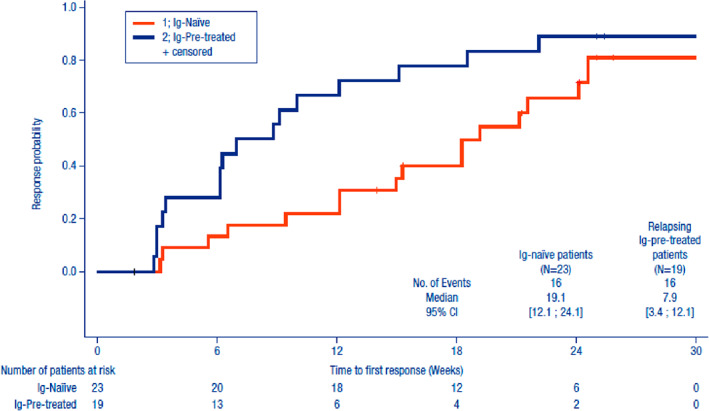
Secondary efficacy endpoint‐time to response (weeks) Kaplan‐Meier curve‐full analysis set (FAS). The Kaplan‐Meier analysis considers responders and non‐responders

The median time to response of responders was 15 weeks with a CI of [8.9‐19.1 weeks]. Although no statistical test was performed, the response seemed to occur earlier in the Ig‐pre‐treated patients with 7.9 weeks with a CI of [3.4‐12.1 weeks] compared to the Ig‐naïve patients with 19.1 weeks with a CI of [12.1‐24.1 weeks]. The Kaplan‐Meier curve is reported in Figure 2. Note that since the first post‐baseline assessment of INCAT disability score was performed 3 weeks after the first course of IqYmune, the first opportunity to identify a response could not arise before 3 weeks (even in patients who improved sooner).

##### Changes in neurological scores and grip strength from baseline to EOS visit

Results for all other neurological scores and for grip strength are presented in Table 3.

The median change from baseline to EOS visit of the adjusted INCAT score was −1.0 point with a 95% CI of [−1.5 to −1.0 points] which is highly statistically significant (*P* < .0001).

All other scores and grip strength measurements showed a statistically significant improvement at EOS visit compared to baseline, supporting the result from the primary efficacy analysis. (Table 4)

**TABLE 4:** Secondary endpoints‐change from baseline to EOS visit in neurological scores and grip strength‐FAS (overall, N = 42)

Parameters	Number of patients	Mean ± SD	Min‐max	Hodges‐Lehmann median (95% CI)^a^	Wilcoxon signed‐rank test^b^
Change in adjusted INCAT disability score *Range: 0 (no disability) to 9 (maximal disability)*	42	−1.2 ± 1.28	−5‐1	−1.0 [−1.5; −1.0]	*P* < .0001
Change in R‐ODS score *Range: 0 (most severe limitations) to 48 (no limitations)*	42	4.0 ± 8.5	−12‐32	3.5 [1.0; 6.0]	*P* = .0036
Change in MRC sum score *Range: 0 (total paralysis) to 60 (normal muscle strength)*	41	3.48 ± 5.8	−11‐18	3.0 [2.0; 5.0]	*P* = .0001
Change in Rasch‐modified MRC sum score *Range: 0 (total paralysis) to 36 (normal muscle strength)*	41	2.8 ± 3.7	−5‐11	2.5 [1.5; 3.5]	*P* < .0001
Change in grip strength in dominant hand *Normalized values (%)* ^*c*^	41	12.0 ± 26.9	−17.8‐109.5	6.2 [1.1; 13.1]	*P* = .0076
Change in grip strength in non‐dominant hand *Normalized values (%)* ^*c*^	41	12.8 ± 29.1	−19.6‐119.1	5.9 [0.2; 14.0]	*P* = .0147

Abbreviations: CI, confidence interval; EOS, end of study; FAS, full analysis set; INCAT, inflammatory neuropathy cause and treatment; MRC, medical research council; R‐ODS, Rasch‐built‐overall disability scale.

^a^ Hodges‐Lehmann median‐unbiased estimate of the population median and associated 95% confidence interval.

^b^ Wilcoxon signed‐rank test using a two‐sided significance level of 5% (difference between the visit and baseline scores did not follow a symmetric distribution around zero).

^c^ Grip strength is reported as the absolute values (in kPa) expressed in percentage of the median value in a healthy population of the same age and gender.^10^

##### Clinical global impression

Based on the investigator assessment, the severity of illness showed that most of the patients were either improved (23/42, 54.8%) or with no change (17/42, 40.5%). Two patients (4.8%) only worsened. In term of global improvement, most of the patients were either improved (35/42, 83.3%) or with no change (3/42, 7.1%). Only four patients worsened (9.5%). In term of efficacy index, most of the patients (33/42, 78.6%) had at least a minimal therapeutic effect without side effect or with side effects interfering not significantly with their functioning. Only one patient had side effects that outweighed the therapeutic effect (minimal in his case).

Patient assessments gave results leading to the same conclusions.

##### Serum total IgG trough levels

At baseline, before the first administration of IqYmune, the mean of serum total IgG trough level was 1070.3 ± 344.5 mg/dL in overall population. After the first administration of IqYmune, the level increased to 1792.0 ± 388.84 mg/dL and then was maintained above 1600 mg/dL until EOS visit. The results were similar in both subgroups.

Logistic regression results did not indicate any relationship between the change from baseline of serum total IgG trough level and the overall clinical response at EOS visit.

### 3.4 Safety

#### 3.4.1 Overall AEs

A total of 311 AEs were observed in 39/43 patients (90.7%) of the TTS including 156 AEs considered as related to IqYmune in 30 patients (69.8%) (supplementary information Table S2). Most drug‐related AEs were mild (136/156; 87.2%), only four were severe. The most common drug‐related AEs were headache (39.5% of patients), pyrexia (16.3% of patients), and myalgia (9.3% of patients). All drug‐related AE resolved without sequelae, except a worsening of a pre‐existing anemia in a patient with gastrectomy treated by vitamin B12. This drug‐related AE did not resolve at EOS visit.

#### 3.4.2 Serious AEs

Nine serious AEs (SAEs) occurred in seven patients from which five were related to IqYmune in three patients (ie, a transient ischemic attack in one patient leading to a premature withdrawal; a severe headache leading to hospitalization, an increase in fibrin D‐dimer and an anaphylactic reaction in one other patient; and a asymptomatic neutropenia in the third patient). All SAEs resolved without sequelae, except a femur fracture, which was recovering at EOS visit.

#### 3.4.3 Biology and vital signs

Results from hematological and biochemistry testing before and after treatment showed:

- No case of renal or hepatic function impairment.
- A decrease in the mean neutrophil count between before and after the first course with two cases of neutropenia reported after the first course of IqYmune that both resolved without sequelae and without corrective medication.

The mean changes in the vital signs (blood pressure, heart rate, and body temperature) from before the course to the end of infusions were unremarkable.

## 4 DISCUSSION

The PRISM study was a prospective, open‐label investigation to assess the efficacy and safety of IqYmune in the initial and maintenance treatment during 6 months in patients with CIDP and to evaluate the role of different scales in the assessment of clinical response. The data showed that, whichever the scale was used for response evaluation, IqYmune can be considered as effective treatment with a favorable benefit‐risk profile in this indication.

Initially this study was planned to complete the European registration dossier of IqYmune after three previous studies assessing the efficacy and safety of this IVIg treatment in PID, ITP, and MMN. This was in line with the 2008 EFNS guidelines for the use of IVIg in treatment of neurological diseases^12^ and the 2010 EFNS/PNS guideline on management of CIDP^9^ that issued a level A recommendation for the use of IVIg as treatment, even as first line in pure motor CIDP. Interestingly, after the PRISM study completion, EMA issued an updated guideline on the clinical investigation of human normal immunoglobulin for intravenous administration,^13^ which came into effect on 1 January 2019. This enabled an approval based on a literature analysis of IVIg in CIDP provided it had been already obtained in PID and ITP. Moreover, the 2019 EMA Guideline on core SmPC for human normal immunoglobulin for intravenous administration added CIDP to the established indications in adults, children, and adolescents (0‐18 years).^14^

Because IVIg is a constraining treatment with repeated infusion courses over a long period, it is interesting to evaluate the compliance at the patient level. A patient was considered as compliant when all of the courses received complied with the following rules: dose prescribed not differing of more than 20% from the theoretical dose according to the protocol; time since previous course within 3 weeks ±7 days and course duration of 2 to 5 days for the first course and 1 to 2 days for the followings; duration of flow rate of the first course infusion remained unchanged for at least 25 minutes; and flow rate of the first infusion of each course was ≤0.5 mL/kg/h. Among a total of 319 courses, only 26 (8.2%) were considered as non‐compliant in 12 of 43 patients (27.9%). This confirmed the good acceptability and feasibility of the treatment.

Regarding the primary endpoint, the 76.2% (95% CI of [60.5%‐87.9%]) of responders with a numerically higher rate in Ig‐pre‐treated patients than in Ig‐naïve patients (84.2% vs 69.6%), lies above the 54% of responders reported in ICE study^3^ after the first 24‐week period. This difference may reflect the different proportion of Ig‐pre‐treated patients in the ICE study (21%) than in PRISM study (46%), since previous responders to IVIg are more likely to respond again to IVIg. However, this does not compromise the results of the primary analysis since the 95% CI of the response rate in Ig‐naïve patients was entirely above 33.3% (historical placebo control group). Our study also differs from ICE study in the period of time given to reach a response: in the ICE study, patients who had not reached a response after 6 weeks were regarded as “non‐responders” and were switched to the other treatment arm; in the PRISM study, treatment response could have occurred at any moment during the 24 weeks treatment period. Therefore, patients had more time to reach a response in the PRISM study than in the ICE study possibly contributing to the higher response rate in PRISM than in the active treatment group of ICE. In addition, the fact that 29% of the patients were still responsive at a later time point (after week 12 and until EOS visit) supports the relevance of the EMA recommendation to administer treatment for a period of 6 months before considering the patient as non‐responder and stopping the treatment.^14^ These results are also in line with the pooled analysis of PRIMA and PATH clinical studies showing that a substantial number of patients (21) become responders after >6 weeks (considered as late responders).^15^

With regard to a possible limitation of the PRISM study to use a historical placebo control group, considering that IVIg has so far demonstrated efficacy in CIDP since the time of ICE study, it was ethically questionable to perform a placebo‐controlled study. In addition, an active control design was not selected due to the weak feasibility of showing a non‐inferiority of IqYmune compared to other products, considering the high number of patients required and the rare population of CIDP. Since our efficacy results were compared to a historical placebo group from the ICE study, some measures to minimize potential bias were considered at the design stage. Inclusion/exclusion criteria were similar in order to avoid selection bias; both Ig‐naïve patients and Ig‐pre‐treated patients could be enrolled. The dose and treatment schedule were the same: 2 g/kg at the initial course followed by 1 g/kg every 3 weeks for the subsequent courses. The primary efficacy endpoint was the same: the responder rate based on a decrease of ≥1 point in the adjusted INCAT disability score.

Despite all the above limitations, all other scores used in PRISM confirmed a positive satisfying effect, supporting the reliability of the results of our study. Similarly, the sensitivity analysis using the PPS confirmed the robustness of the primary analysis with a response rate of 73.5% (95% CI 55.6%‐87.1%) similar to the FAS.

When reviewing results of the PRISM study in detail, Ig‐pre‐treated patients showed a numerical trend for a higher responder rate than Ig‐naïve patients (84.2% vs 69.6%) possibly reflecting the fact that Ig‐pre‐treated patients were selected for their positive clinical response to IVIg and consequently are more likely to respond again. This may also explain the earlier response in Ig‐pre‐treated patients than in Ig‐naïve patients as also observed in a previous study.^4^

The decrease in the mean adjusted INCAT score change between baseline and EOS visit was also higher in the Ig‐pre‐treated patients than in the Ig‐naïve patients (−1.6 ± 1.21 points vs −0.9 ± 1.28 points). This may reflect the fact the Ig‐pre‐treated patients started from a mean higher score value (3.4 ± 1.77 points vs 2.7 ± 1.26 points), possibly due to their inclusion in the trial at the time of relapse, but ended up with a comparable score at EOS (1.7 ± 1.19 points vs 1.8 ± 1.65 points).

In this study, we also found a good correlation between the investigator and the patient assessment of response to treatment. Among the 78 assessments performed in 40 patients by both patient and investigator at week 12 or EOS visit, 58 (74%) were similar between the patients and the investigators, in 9 (12%), the patients were less satisfied than the investigators and in 11 (14%), the patients were more satisfied than the investigators.

We did not find any correlation between the clinical response and the IgG level variations between before and 14 days after the maintenance dose.^16^ It was previously reported that the increase in serum IgG level after a standard dose of IVIg during a stable maintenance treatment of CIDP is relatively constant within individual patients, but differs between patients treated with the same IVIg dose and treatment schedule^17^ without a clear‐cut correlation with clinical response. A better understanding of the reasons for this heterogeneity between patients might help however to better individualize therapy with IVIg.^18^

The safety is in line with the use of IVIg in CIDP. Regarding headaches, they were also the most frequent drug‐related AEs reported in previous clinical studies.^3^, ^4^ They are well known adverse reactions to IVIg and may be related to the rate of infusion.^14^ It is interesting to note that, in the patient experiencing three SAEs, the headache appeared on the fourth day of the first treatment course, 20 minutes after the flow rate had been increased to 4 mL/kg/h and was initially moderate. However, the infusion flow rate was nevertheless increased to 6 mL/kg/h until the course completion and 30 minutes after the end of infusion, the headache became severe leading to hospitalization (first SAE). In the context of recurrent headache in this patient, further investigations were performed. An increase in fibrin D‐Dimer up to 6000 μg/L was reported (second SAE in this patient). Based on these results, a CT scan and angio‐MRI were performed and ruled out a cerebral venous thrombosis. It can be hypothesized that avoiding a dose increase in front of this headache would have prevent its worsening. Same, at the time of the fifth treatment course, the course had been prescribed over a single day (when previous were over 2 days), and started at an infusion flow rate of 6 mL/kg/h (instead of 0.5 mL/kg/h as recommended in the protocol) which could explain the poor tolerance with a blood pressure fall, fever and tachycardia diagnosed as an anaphylactic reaction by the investigator (third SAE in this patient). The small number of thromboembolic events in our study probably reflects the exclusion of patients with risk factors for these events possibly limiting the external validity of this finding considering their relatively high frequency in the adult population.

In conclusion, the PRISM study demonstrated that IqYmune administered as a regimen of a 2 g/kg induction dose and 1 g/kg maintenance doses every 3 weeks is an effective treatment in patients with CIDP with a superiority of IqYmune compared to the historical placebo control after 24 weeks of treatment in CIDP. In addition, no new safety finding or trend arised from this study confirming the known safety profile of this group of therapies.^15^

## Supporting information


**Table S1** Patient exposure‐TTS
**Table S2** Adverse events reported in ≥5% of patients‐TTS (Overall, N = 43)
