# Supplementary material for: An international multicenter efficacy and safety study of IqYmune in initial and maintenance treatment of patients with chronic inflammatory demyelinating polyradiculoneuropathy: PRISM study
Source: J Peripher Nerv Syst. 2020 Aug 31;25(4):356–65. doi: 10.1111/jns.12408 (PMC7754365; doi:10.1111/jns.12408)
Supplement: Supplementary file 1 — Table S1 Patient exposure‐TTS Table S2 Adverse events reported in ≥5% of patients‐TTS (Overall, N = 43) [file JNS-25-356-s001.docx]

**Supplementary tables**

**Table S1.** Patient exposure – TTS

|  | **Ig-naïve N=23** | | **Ig-pre-treated N=20** | **Overall N=43** |
| --- | --- | --- | --- | --- |
| **Duration of IqYmune^®^ exposure (months)** | | |  | |
| Mean ± SD  Median  Min – Max | 5.6 ± 0.6  5.7  3.2 – 6.1 | | 4.9 ± 1.8  5.6  0.2 – 5.9 | 5.3 ± 1.4  5.7  0.2 – 6.1 |
| **Total number of courses** |  | |  |  |
| Mean ± SD  Median  Min – Max | 7.8 ± 0.7  8.0  5 – 8 | | 7.0 ± 2.5  8.0  1 – 8 | 7.4 ± 1.8  8.0  1 – 8 |
| **Total number of infusions** |  | |  |  |
| Mean ± SD  Median  Min – Max | 14.6 ± 4.0  17.0  8 – 19 | | 12.3 ± 5.8  10.5  2 – 19 | 13.5 ± 5.0  16.0  2 – 19 |
| **Maximal flow rate over all infusions (mL/kg/h)** | |  | | |
| Mean ± SD  Median  Min – Max | 3.6 ± 1.7  2.9  1.8 – 6.0 | | 3.3 ± 1.7  2.0  2.0 – 6.0 | 3.4 ± 1.7  2.7  1.8 – 6.0 |

Abbreviations: N, number of patients; SD, standard deviation; TTS, total treated set.

**Table S2.** Adverse events reported in ≥5% of patients – TTS (Overall, N=43)

|  | **All AEs** | | | **Drug-related AEs** | |
| --- | --- | --- | --- | --- | --- |
|  | | **Number (%) of patients** | **Number of AEs** | **Number (%) of patients** | **Number of AEs** |
| Any AEs | | 39 (90.7) | 311 | 30 (69.8) | 156 |
| Headache | | 20 (46.5) | 80 | 17 (39.5) | 62 |
| Pyrexia | | 8 (18.6) | 11 | 7 (16.3) | 8 |
| Influenza | | 8 (18.6) | 10 | 0 | 0 |
| Nausea | | 5 (11.6) | 6 | 3 (7.0) | 4 |
| Myalgia | | 5 (11.6) | 5 | 4 (9.3) | 4 |
| Urinary tract infection | | 5 (11.6) | 5 | 0 | 0 |
| Arthralgia | | 4 (9.3) | 6 | 2 (4.7) | 3 |
| Asthenia | | 4 (9.3) | 4 | 3 (7.0) | 3 |
| Chills | | 3 (7.0) | 10 | 3 (7.0) | 10 |
| Vertigo | | 3 (7.0) | 5 | 2 (4.7) | 3 |
| Malaise | | 3 (7.0) | 5 | 3 (7.0) | 5 |
| Pruritus | | 3 (7.0) | 4 | 2 (4.7) | 2 |
| Rash | | 3 (7.0) | 3 | 3 (7.0) | 3 |
| Anaemia | | 3 (7.0) | 3 | 1 (2.3) | 1 |
| Oropharyngeal pain | | 3 (7.0) | 3 | 0 | 0 |

Abbreviations: AEs, Adverse events; TTS, total treated set.
